# Caffeine-Induced Sleep Restriction Alters the Gut Microbiome and Fecal Metabolic Profiles in Mice

**DOI:** 10.3390/ijms232314837

**Published:** 2022-11-27

**Authors:** Zan Song, Lin Liu, Yanyi Xu, Ruofan Cao, Xianyong Lan, Chuanying Pan, Shengxiang Zhang, Haiyu Zhao

**Affiliations:** 1 School of Life Sciences, Lanzhou University, No. 222 South Tianshui Road, Lanzhou 730000, China; 2 Key Laboratory of Animal Genetics, Breeding and Reproduction of Shaanxi Province, College of Animal Science and Technology, Northwest A&F University, No. 22 Xinong Road, Yangling, Xianyang 712100, China

**Keywords:** caffeine consumption, sleep restriction, mice, gut microbiome, microbiota, fecal metabolome

## Abstract

Insufficient sleep is becoming increasingly common and contributes to many health issues. To combat sleepiness, caffeine is consumed daily worldwide. Thus, caffeine consumption and sleep restriction often occur in succession. The gut microbiome can be rapidly affected by either one’s sleep status or caffeine intake, whereas the synergistic effects of a persistent caffeine-induced sleep restriction remain unclear. In this study, we investigated the impact of a chronic caffeine-induced sleep restriction on the gut microbiome and its metabolic profiles in mice. Our results revealed that the proportion of *Firmicutes* and *Bacteroidetes* was not altered, while the abundance of *Proteobacteria* and *Actinobacteria* was significantly decreased. In addition, the content of the lipids was abundant and significantly increased. A pathway analysis of the differential metabolites suggested that numerous metabolic pathways were affected, and the glycerophospholipid metabolism was most significantly altered. Combined analysis revealed that the metabolism was significantly affected by variations in the abundance and function of the intestinal microorganisms and was closely relevant to *Proteobacteria* and *Actinobacteria*. In conclusion, a long-term caffeine-induced sleep restriction affected the diversity and composition of the intestinal microbiota in mice, and substantially altered the metabolic profiles of the gut microbiome. This may represent a novel mechanism by which an unhealthy lifestyle such as mistimed coffee breaks lead to or exacerbates disease.

## 1. Introduction

Sleep is an essential part of life for most organisms. All mammals spend a large proportion of their lives asleep to maintain physical and mental health. However, insufficient sleep characterizes our modern society, and an increasing number of people are sleep-deprived because of extended working hours or increased social demands. Inadequate sleep impacts organisms in numerous ways, for instance, leading to cognitive, immunologic, and metabolic deficits [1,2,3]. A short-term sleep deprivation can lead to difficulties in regulating one’s mood and emotion, as well as the cognitive functions, while in the long-term, sleep deprivation can lead to neurological disorders, including depression [4]. Non-specific immune parameters are activated when sleep is restricted, and both a chronic sleep restriction and sleep deprivation for only one night can impair the formation of an antigen-specific immune defense, as reflected by the antibody production and humoral immune response [5]. In addition, from a metabolic point of view, a chronic sleep deficiency has been closely associated with metabolic diseases, including obesity and diabetes; even acute periods of a sleep restriction may result in the alteration of the glucose metabolism, hormone production, and amino acid levels [6,7,8,9,10,11].

Cases of sleep loss are commonly linked to the consumption of caffeinated beverages, which are increasingly relied upon to maintain alertness and improve attention. Caffeine (1,3,7-trimethylxanthine) is the most widely used psychostimulant, and it can enhance the excitability of the adenosine-sensitive sympathetic nervous system, thus impacting both sleep and circadian physiology [12,13]. Over the years, the efficacy of caffeine as a countermeasure for sleepiness has been well-established [14,15,16,17]. It is worth noting that a moderate caffeine intake can combat sleep deprivation and increase alertness and concentration, while an excessive caffeine consumption increases sleep pressure and decreases sleep quality [18,19]. Therefore, people should consume caffeine in moderation and, importantly, at an appropriate time. However, the truth is, cycles of caffeine consumption and insufficient sleep generally tend to succeed each other.

Mammals possess diverse and active gut microbiota, which are readily affected by environmental factors and their physiological state [20,21]. Insufficient sleep results in various pathological states in conjunction with gut dysbiosis, and a number of studies have investigated the gut microbiome in the context of sleep disruption in humans as well as in other model systems, using heterogeneous protocols to simulate either an acute sleep deprivation or chronic sleep fragmentation [22,23,24,25]. In addition, a caffeine consumption may have health consequences which could depend in part on alterations of the gut microbiome. For example, due to its antimicrobial properties, caffeine might affect redox homeostasis, or even induce gut dysbiosis [26,27]. Moreover, caffeine consumption has been clinically correlated with preventive effects against several pathologies, with the amplification of certain bacterial populations being beneficial to the health of the host [28].

It has been well-documented that changes in the gut microbiota may affect the physiology of the host organism, including effects on metabolic processing, immunity, and behavior [29,30,31]. Microbes produce various chemicals, hormones, and vitamins, and therefore are closely related to the metabolism of the host [32,33]. Previous studies have demonstrated that a chronic sleep restriction results in the widespread disturbance of metabolic profiles in the brain, serum, and urine [34,35]. The consumption of caffeine has been associated with reducing the risk of metabolic syndrome and the enhancement of insulin resistance [36,37,38]. In addition, it has been also demonstrated to alter the metabolism of carcinogens, thereby reducing the risk of cancer [39]. Even though the pharmacological effects of caffeine, the adverse impact of sleep restriction on organisms, and their respective effects on the gut microbiota have been widely investigated, few systematic studies have been conducted to elucidate the long-term effects of a daily caffeine-induced sleep restriction on the gut microbiota and metabolism of the host organism [2,39,40,41]. In the present study, to simulate human daily activities, we restricted the sleep of mice during the dark period to mimic the human active period and administered caffeine orally during the early light period to mimic the consumption of caffeine at their early sleep stages. We applied an integrated approach combining full-length 16S rRNA sequencing and a liquid chromatography-mass spectrometry (LC-MS)-based metabolomic analysis, aiming at elucidating the effects of a chronic caffeine-induced sleep restriction (CISR) on the diversity of the gut microbiome as well as their metabolic profiles.

## 2. Results

### 2.1. CISR Model and the Workflow to Study Functional Changes in the Gut Microbiome

**Figure 1:**
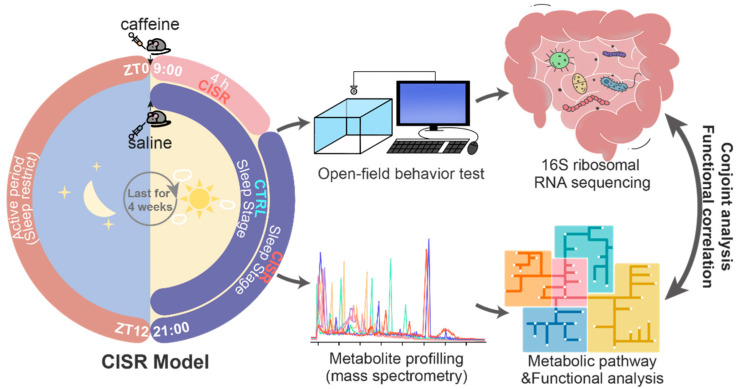
Schematic diagram of the caffeine-induced sleep restriction (CISR) model and the workflow of this study. An integrated approach combining 16S rRNA gene sequencing and liquid chromatography-mass spectrometry (LC-MS)-based metabolite profiling to explore the functional impact of chronic CISR on the gut microbiome as well as its metabolome in a mouse model. ZT in this figure refers to the zeitgeber time.

In this study, we established a 4-week CISR research model in C57BL/6N mice to simulate the daily activities of humans, as schematically illustrated in Figure 1. To verify that the 4 weeks of caffeine gavage efficiently induced a sleep restriction in the mice, their spontaneous movement was randomly recorded 1 h after the saline or caffeine gavage (precisely from 10:00 to 13:00). The results showed that the mice in the CISR group were clearly more active, as indicated by a significant increase in the behavioral parameters compared to the CTRL group, including the distance moved, average velocity, and active time (Figure S1).

Following 4 weeks of CISR, 16S rRNA gene sequencing and LC-MS were carried out to determine the impact of CISR on the gut microbiome and its metabolic profiles (Figure 1). Briefly, DNA was isolated from fecal pellets, amplified by a polymerase chain reaction (PCR) using 16S rRNA primers, followed by full-length sequencing using the Pacific Biosciences Sequel II System platform (Menlo Park, CA, USA) (Table S1). The sequencing results were further processed by using the Qiime software (version 1.8.0) to reveal CISR-induced gut microbiome changes. Next, fecal metabolites were extracted and analyzed by using a mass spectrometry-based metabolome analysis. Molecular features were processed and analyzed with the XCMS software to profile metabolites that showed significant changes between the CTRL and CISR animals. The resultant peak list with the exact mass was subsequently searched against the Human Metabolome Database (HMDB, Version 5.0). The matched exact mass and associated retention time were used to generate the MS/MS spectra to confirm the identities of the various metabolites, followed by the metabolic pathway and functional analysis with the KEGG and HMDB databases. Finally, the correlations between the altered gut microbiome and shifted metabolome were conjointly analyzed to reveal the functional impacts of CISR on the gut microbiome.

### 2.2. Chronic CISR Induced Gut Microbiome Changes

**Figure 2:**
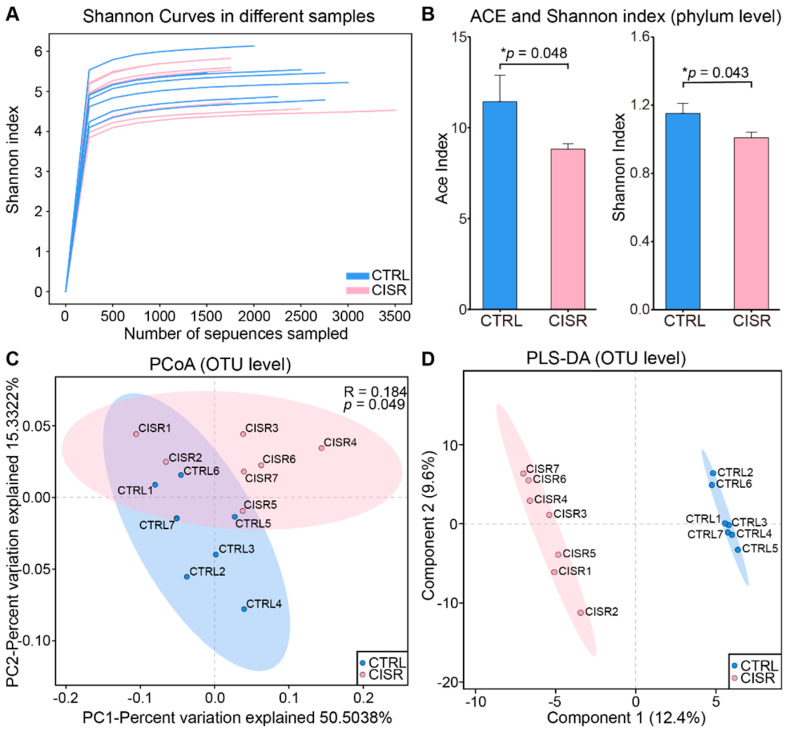
α and β diversity analysis of gut microbiota showed differences between the CTRL and CISR groups. (**A**) The Shannon index curves of all samples. (**B**) Comparison of ACE (abundance-based coverage estimators) and Shannon index between CTRL and CISR groups at the phylum level. The values were presented as the mean ± SEM. Significant differences were indicated by asterisks (Wilcoxon rank-sum test, * *p* < 0.05). (**C**) A principal coordinate analysis (PCoA) based on a weighted UniFrac analysis of the CTRL and CISR samples. (**D**) A partial least squares discriminant analysis (PLS-DA) indicated that CTRL and CISR samples clustered in separate groups.

To verify the accuracy of 16S rRNA sequencing and to evaluate the diversity of the gut bacteria in all the samples, Shannon index curves were constructed based on individual samples, and all these curves reached the saturation plateau (Figure 2A), suggesting that our sequencing could capture the complete diversity of communities among the samples.

To further distinguish the differences between the CTRL and CISR groups, the ecological characteristics were evaluated by using various indices from OTU to the phylum level. At the phylum level, CISR significantly reduced both the abundance-based coverage estimators (ACE) and Shannon indexes (*p* < 0.05, Figure 2B), whereas no significant difference was observed at the other levels. Furthermore, the differences in the intestinal microbiome patterns arising from the CISR was readily differentiated by using a multivariate statistical analysis, as shown by the PCoA plots (Figure 2C). The CTRL and CISR animals were well-separated, with 50.50% and 15.33% variation explained by principal components 1 and 2, respectively. The subsequent analysis of similarities (ANOSIM) test showed that the overall microbial diversity differed between these two groups (R = 0.184, *p* = 0.049). Consistent with the PCoA plot, the beta diversity analysis via the partial least squares discriminant analysis (PLS-DA) demonstrated that there was indeed a great difference between the two groups (Figure 2D). These results suggested that CISR significantly altered the diversity of the gut microbiota in mice, and especially reduced the alpha diversity at the phylum level.

### 2.3. Differentially Abundant Bacterial Groups in Mice following CISR

**Figure 3:**
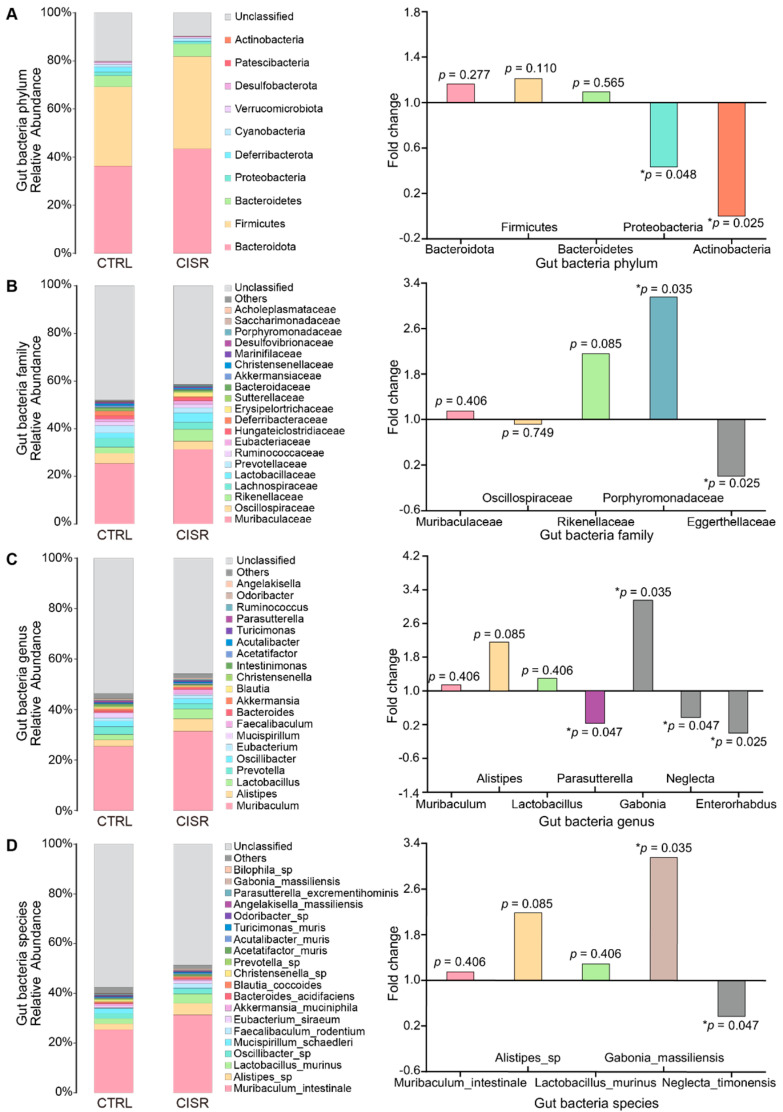
Quantitative visualization of microbial composition and the significantly altered microflora. Species abundance distribution map of the CTRL and CISR groups at the (**A**) phylum, (**B**) family, (**C**) genus, and (**D**) species levels. Wilcoxon rank-sum test of variance between groups showed altered gut microbiota composition at these levels. The fold change (FC) values and the up-down regulation of the flora with greater abundance or significant differences were shown in the right panels. The values were presented as the mean ± SEM. Significant differences were indicated by asterisks (* *p* < 0.05).

We next examined whether the gut microbiota with commonly assigned taxonomic ranks were significantly modulated during chronic CISR (Figure 3 and Table S2). At the phylum level, the abundance of *Bacteroidota* (26.59–51.49%), *Firmicutes* (28.91–57.17%), and *Bacteroidetes* (2.05–8.09%) accounted for a relatively large proportion of all samples, while no significant differences in these phyla was detected between the CTRL and CISR groups. Interestingly, at the phylum level, the abundance of *Actinobacteria* and *Proteobacteria* were significantly reduced after the CISR treatment (*p* < 0.05, Figure 3A).

At the family level, the relative abundances of *Muribaculaceae* (13.40–42.84%), *Oscillospiraceae* (1.53–7.46%), and *Rikenellaceae* (1.62–10.74%) were predominant in both the CTRL and CISR groups (Figure 3B). *Porphyromonadaceae* was more prominently present in the CISR group, whereas *Eggerthellaceae* was more abundant in the CTRL group (*p* < 0.05). *Muribaculum* (13.40–42.84%), *Alistipes* (1.62–10.74%), and *Lactobacillus* (0.09–8.12%) were found to be more abundant at the genus level in both the CTRL and CISR groups, and again no significant difference was observed at these genera upon CISR treatment (*p* > 0.05). However, our results revealed that even though present at a relatively lower abundance, the *Enterorhabdus*, *Neglecta*, and *Parasutterella* were significantly reduced, while *Gabonia* tended to be more abundant in the CISR group (*p* < 0.05) (Figure 3C). Interestingly, *Muribaculum_intestinale* (13.40–42.84%), and *Alistipes_sp.* (1.43–10.47%) were the most abundantly detected microbial species, while only the abundance of *Gabonia_massiliensis* and *Neglecta_timonensis* was significantly altered during the CISR treatment (*p* < 0.05) (Figure 3D).

**Figure 4:**
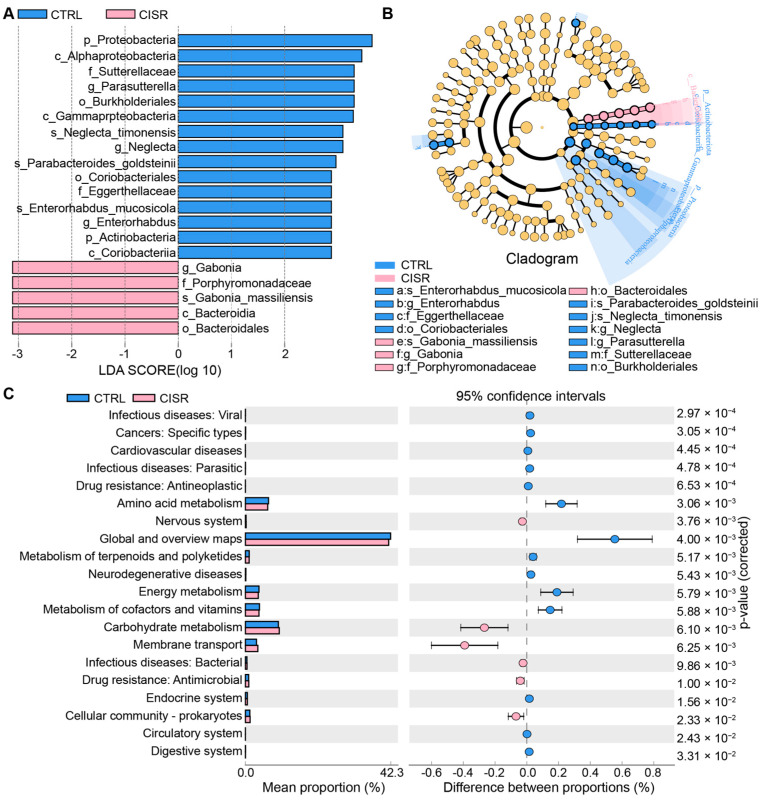
Distinct taxa with greater contributions at different taxonomic levels and the predicted functional changes in the gut microbiota. (**A**) Linear discriminant analysis (LDA) effect size (LEfSe) identified microbial biomarkers from phylum to species levels that discriminate the CTRL and CISR groups. (**B**) Cladogram constructed using the LEfSe method to display the phylogenetic distribution of bacteria that were most highly enriched in the CTRL and CISR groups. Nodes in red and blue indicate the significant taxa. (**C**) The KEGG analysis showed the potential pathways associated with changes in the intestinal microbiota after the CISR treatment (*p* < 0.05).

A linear discriminant analysis LDA effect size (LEfSe) was performed to identify the taxa most likely to explain differences in the composition of the microbiota between the CTRL and CISR animals. A comparison of the individuals from both groups revealed that Proteobacteria and Actinobacteria at the phylum level, as well as *Parasutterella*, *Neglecta,* and *Enterorhabdus* at the genus level exhibited significant influences in the CTRL group, while in the CISR group, *Gabonia* at the genus level and *Porphyromonadaceae* at the family level made contributions (Figure 4A). A taxonomic cladogram derived from the LEfSe analysis (with the LDA score > 2.0) showed the relationships of the differential microbiota with significant roles (Figure 4B). Altogether, these results suggest that the gut microbiota exhibit alterations in their composition following a chronic caffeine-induced sleep restriction.

A KEGG functional prediction was subsequently performed at the genus level by means of the PICRUSt approach. The results showed that these differential microbes might be closely related with energy and carbohydrate metabolism, digestive and nervous systems, as well as the metabolism of amino acids, terpenoids, polyketides, cofactors, and vitamins (Figure 4C). Based on all these predictions, we next investigated the metabolic functional changes in intestinal microbiota in response to CISR by examining the actual fecal metabolic profiles of the mice.

### 2.4. CISR Induced Significant Metabolic Changes in the Gut Microbiome

**Figure 5:**
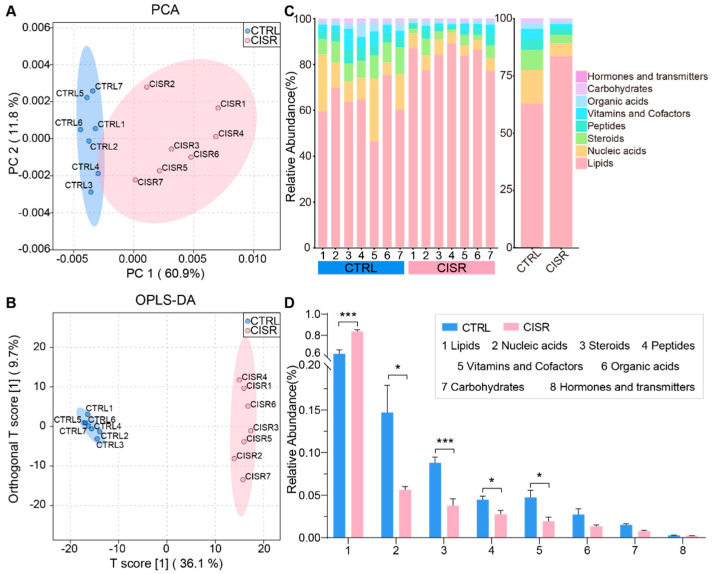
CISR perturbed the fecal metabolic profiles. (**A**,**B**) The fecal metabolite profiles of the CTRL and CISR mice differentiated by a principal component analysis (PCA) and an orthogonal partial least squares discrimination analysis (OPLS-DA). (**C**,**D**) CISR altered the relative abundance of all fecal metabolites, as shown by a percentage stacking histogram and a comparison via Student’s *t*-test. The values were presented as the mean ± SEM. Significant differences were indicated by asterisks (*** *p* < 0.001, * *p* < 0.05).

The combination of a large quantity of gut bacteria and their high concentrations of metabolic products in feces confers ideal samples to assess the functional changes in the gut microbiome. Thus, to further investigate whether CISR disrupts the intestinal metabolism, LC-MS/MS was used to detect the metabolite components in each fecal sample. The first two components identified by the PCA were used to evaluate the intestinal metabolic changes between the CTRL and CISR mice (Figure 5A). Furthermore, an orthogonal partial least squares discrimination analysis (OPLS-DA) model of their metabolic patterns was also established (Figure 5B). The samples of these two groups could be significantly differentiated and well-separated by both analytical models, indicating that the intestinal metabolism of mice could be remarkably altered after the chronic caffeine disturbance of their sleep status.

**Figure 6:**
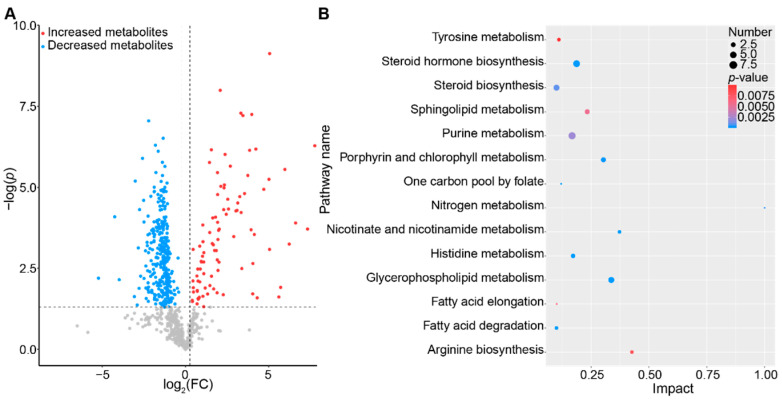
Key metabolic pathways perturbed by CISR. (**A**) CISR perturbed the metabolic profiles of fecal samples in mice, with 405 metabolites significantly changed in comparison with the normal CTRL group (fold change > 1.2, *p* < 0.05). (**B**) Pathway enrichment analysis of differentially expressed metabolites.

The stacking histograms were plotted according to the identified metabolites in different samples, with the abundance percentages of each type of metabolites shown (Figure 5C). Of all the identified metabolites, lipids accounted for the largest proportion and were significantly up-regulated during the chronic CISR treatment (*p* < 0.001), while the other metabolites, which were categorized as nucleic acids, steroids, peptides, vitamins, and cofactors, were all significantly down-regulated (*p* < 0.05 or *p* < 0.001, Figure 5D). Thus, the results suggest that chronic CISR strongly altered the fecal metabolic profiles, with lipids increased exclusively while the other metabolites decreased. Specifically, a total of 405 annotated metabolites with greater than 1.2-fold changes between the CTRL and CISR groups were identified (Figure 6A and Figure S2, Table S4), of which 93 metabolites were significantly up-regulated while 312 were down-regulated (*p* < 0.05). Furthermore, to identify the key pathways in which the differential metabolites were enriched, a pathway analysis was performed by using the Metaboanalyst (https://www.metaboanalyst.ca/, version 5.0, accessed on 16 June 2021) (Figure S3, Table S5). The topological influence of the KEGG pathways was calculated, and the impacts of these pathways on the observed metabolic changes were evaluated. Our results revealed that there were fourteen metabolic pathways associated with changes in the intestinal metabolism in the mice upon the CISR treatment (impact > 0.1, *p* < 0.05, Figure 6B), for instance, the glycerophospholipid metabolism, steroid hormone biosynthesis, fatty acid degradation, purine metabolism, sphingolipid metabolism, fatty acid elongation, and tyrosine metabolism. Among all these identified pathways, the glycerophospholipid metabolism was the most noteworthy since it was the most critical and significantly altered metabolic pathway (ORA, *p* = 2.42 × 10^−6^, impact = 0.33728) identified by the correlation and metabolomics pathway analysis.

### 2.5. Associations between the Gut Microbiome and Fecal Metabolic Profiles

**Figure 7:**
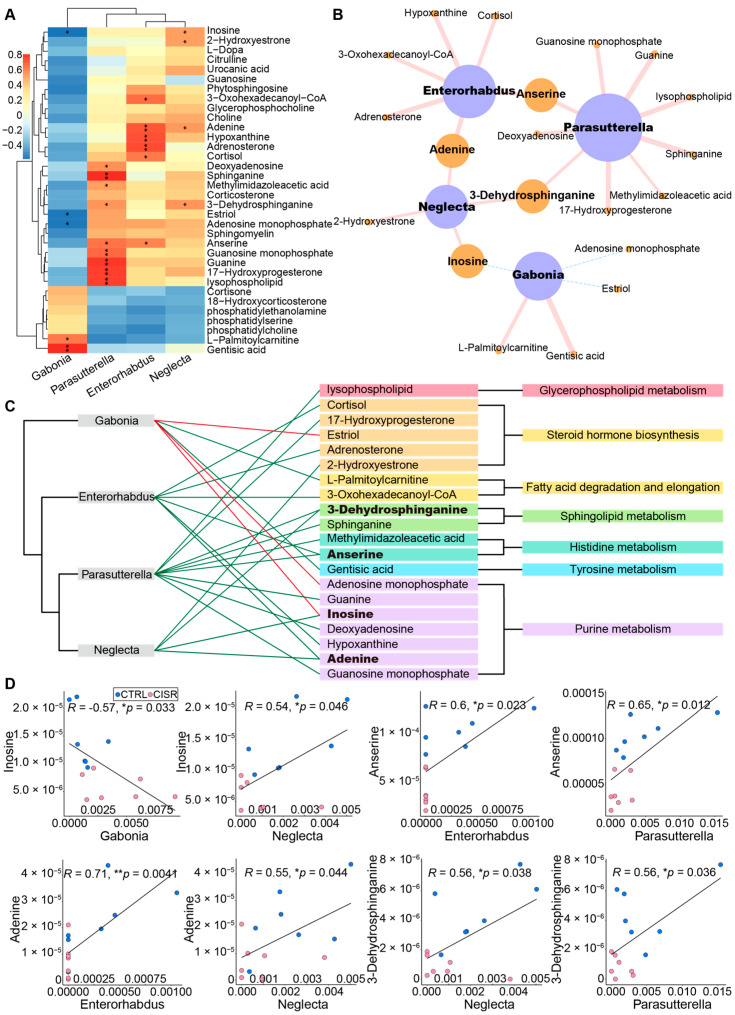
CISR induced metabolic changes correlated with differences in the gut microbiome. (**A**) Correlation plot showed the functional correlation between perturbed gut bacteria and altered fecal metabolites at the genus level. (**B**,**C**) An integrated correlation-based network analysis (Pearson’s correlation) of microbes and metabolites. (**B**) The size of the nodes represented the number of significant correlations, and the thickness of lines represented the strength of correlations. The pink solid lines and a blue dotted lines represented positive and negative correlations, respectively. (**C**) The direction of the correlation was indicated by green (positive) or red (negative) lines, and bold indicates metabolites with greater correlativity. (**D**) Scatter plots illustrate associations between altered gut bacteria genera and typical gut microflora-related metabolites, including glycerophospholipids, fatty acids, amino acids, and purines (* *p* < 0.05; ** *p* < 0.01, Pearson’s test).

To further explore the functional correlation between the changes in the gut microbiome and the fecal metabolic profiles, a Pearson’s correlation matrix was generated by calculating the coefficient between the four genus-level differential microflora and all differentially abundant metabolites of the CTRL and CISR groups (Figures S4 and S5). We detected correlations between these four microflora and distinct metabolites (34 compounds mainly involved in the glycerophospholipid, purine, sphingolipid, histidine, and tyrosine metabolism pathways, steroid hormones, and the arginine biosynthesis pathway, *p* < 0.05) (Figure 7A) in particular. Significant associations were selected (*p* < 0.05), and their biological networks were visualized by using the Cytoscape (version 3.5.1, Seattle, WA, USA). Based on these visualizations, clear correlations were identified between the perturbed gut microbiome and the altered metabolite profiles (Figure 7B). Specifically, two microbial species played a major metabolic role and were associated with 70% of the differential pathway-related metabolites: *Parasutterella* (nine metabolites) and *Enterorhabdus* (six metabolites). Among the distinct metabolites, anserine, adenine, inosine, and 3-dehydrosphinganine had two correlations with different microbial species. They were derived from the sphingolipid metabolism, histidine metabolism, and purine metabolism, respectively (Figure 7C). To demonstrate the functional correlation between the gut microbiome and fecal metabolites, we showed these four metabolites that were strongly associated with genus-level differential gut bacteria (Figure 7D). For example, adenine and inosine, associated with the purine metabolism, were positively correlated with the *Parasutterella* and *Enterorhabdus*. Anserine and 3-Dehydrosphinganine were assigned to the histidine metabolism and sphingolipid metabolism, respectively. The former is positively correlated with *Parasutterella* and *Enterorhabdus*, and the latter is positively correlated with *Neglecta* and *Parasutterella*. In conclusion, the physiological effects of CISR on mice might be realized by changes in the microbial metabolism. The analysis of different microbial metabolites and pathways revealed that the altered levels of differential metabolites could be significantly associated with Proteobacteria (p_*Proteobacteria*; g_*Parasutterella*) and Actinobacteria (p_*Actinobacteria*; g_*Enterorhabdus*).

## 3. Discussion

Currently, the consumption of caffeine to maintain wakefulness followed by CISR is very common, and is particularly prominent among specific groups [42,43,44]. Research has verified that changes in intestinal microbes are closely related to human health, but these changes are susceptible to certain factors including individual circadian rhythms [45]. In this study, we compared mice on a regular sleep schedule and those subjected to CISR to explore its effects on the intestinal microbiota. Our result revealed that CISR perturbed the diversity and composition of the gut microbiome and distinctly changed its metabolism and function of the gut microbiota. We showed that the most abundant metabolites identified were lipids and the most significantly altered metabolic pathway was the glycerophospholipid metabolism. Importantly, we found that differential metabolites were more relevant to the changes in *Proteobacteria* and *Actinobacteria*. Our results offered possible explanations for the widespread health concerns about the effects of sleep loss on intestinal health caused by a caffeine consumption and they raise important questions about precisely how metabolic changes associated with gut microbiome perturbations can be induced by the ill-timed consumption of coffee in daily life.

### 3.1. Chronic CISR Affects Gut Microbiota in Mice

The effects of sleep loss on gut microbial diversity and composition are perennial topics. Sleep deprivation disrupts the gut microbiota, negatively affects the gut permeability, and enhances the translocation of viable bacteria from the gut [46]. The gut microbiome affects sleep by altering the balance of neurotransmitters in the gut [47]. In addition, sleep deprivation increases the risk of depression, which in turn increases the risk of insomnia [48,49]. Moreover, the gut microbiome regulates host sleep and mental states through the microbiome–gut–brain (MGB) axis [50]. In summary, there is a network of relationships between gut microbiome, sleep deprivation, and depression, and the gut microbiome is indispensable for the research and treatment of human mental disease and sleep disorders. Research has shown that a long duration of sleep deprivation may lead to changes in α-diversity, while β-diversity is usually not affected [46]. However, we found more significant changes in the microbial community profiling, with a significant separation between the control and CISR groups in the β-diversity analysis, and a significant decrease in the ACE index in the alpha diversity index after the CISR treatment. We also found a significant decrease in the abundance of *Proteobacteria* and *Actinobacteria*. Various studies have suggested that *Proteobacteria* is a Gram-negative bacteria that is commonly overrepresented in several intestinal diseases and causes a decrease in intestinal mucus, which triggers the impaired intestinal barrier and low-grade inflammation [51,52]. In our results, similar to the previous studies, the abundance of pro-inflammatory bacteria *Proteobacteria* was significantly decreased after the CISR treatment, suggesting that a sleep restriction may not induce gut dysbiosis and inflammatory responses through this microbial pathway [53]. *Actinobacteria* is widely used as probiotics, demonstrating beneficial effects on numerous pathological conditions, and they are critical in the maintenance of gut homeostasis, the modulation of gut permeability, the immune system, and gut–brain axis [54]. However, the abundance of probiotic *Actinobacteria* was significantly decreased after CISR, which was primarily related to the disruption of gut dysbiosis by a sleep restriction [46].

In this study, we did not observe significant changes in the F/B (*Firmicutes*/*Bacteroidetes*) ratio. Numerous studies have found an increased F/B ratio in the gut microbiota in both mice and humans subjected to a sleep disruption and restriction [22,24,55]. However, a caffeine consumption could attenuate the increase in the F/B ratio in high-fat-fed rats [38]. Caffeine has been demonstrated to influence the communication between bacteria as a potential quorum sensing inhibitor of bacterial pathogenesis, with some antioxidative and anti-inflammatory properties, as well as antimicrobial effects [56,57,58]. In addition, caffeine has complex and dose-dependent effects on gut immunity. For example, caffeine has been demonstrated to exhibit anti-inflammatory effects at low concentrations and pro-inflammatory effects at high levels by inhibiting or activating cytokines [59]. As for the role of caffeine in sleep restriction, it has been suggested to exert a neuroprotective effect, meliorate the dysbiosis of gut microbiota, and reduce the levels of proinflammatory factors in serum [53,60]. Considering the above effects, we do not deny the possible beneficial effects of caffeine, but the inopportune use of caffeine in our CISR model caused negative effects. All these results suggest that CISR can promote changes in the gut environment, increase the risk of a gut flora perturbation, and affect microbial diversity in mice, but changes in the proportions of the dominant phyla may not represent a core mechanism for the CISR effects on gut health.

### 3.2. Appreciable Impact of CISR on Intestinal Metabolites

To determine whether our CISR paradigm functionally affects the gut microbiome, we measured the species and abundance of fecal metabolites. According to previous studies, sleep deprivation can induce gut microbiota disorders via oxidative stress and inflammatory responses, and contribute to metabolic dysfunction partially through the initiation of physiological stress responses [61,62,63]. Both animal and human studies have shown that sleep loss affects multiple classes of fecal metabolites, including inosine and bile acid, and, interestingly, we observed similar metabolic changes [24,55,64]. In addition, the lipid metabolism has been indicated to be affected by circadian rhythm; the decrease in leptin and increase in the ghrelin levels caused by a sleep deficiency could lead to a weight gain, lipid accumulation, and obesity-related metabolic disorders [62,65]. Additionally, our fecal metabolome analysis revealed that the lipid metabolism was primarily affected, most of the significantly modulated metabolites were related to the glycerophospholipid and sphingolipid metabolism, fatty acid degradation, and elongation pathways. We also identified that the glycerophospholipid metabolism was one of the most critical metabolic pathways. Lipids are highly biologically active metabolites that are involved in a wide range of inflammatory processes [66]. The excretion of higher short-chain fatty acids (SCFAs) has been associated with health problems such as dysbiosis, excess adiposity, and cardiometabolic diseases [67].

A caffeine intake has been linked to weight loss and the regulation of the lipid metabolism, in part through its inhibition of adipogenesis-related factors [68]. Caffeine decreases the lipid accumulation in adipocytes, and causes an increase in lipid metabolites, which is consistent with our results [69]. Other studies have indicated that caffeine inhibits the incorporation of adenine and thymidine in the synthesis of DNA via the inhibition of thymidine kinase; this could be reflected by the changes in purine metabolites and the decline of pro-inflammatory bacterial populations in this study [70,71]. Therefore, we hypothesized that there was a combined effect of caffeine and sleep deprivation after CISR: caffeine could modulate the lipid metabolic pathways in the gut microbiota, such as the glycerophospholipid metabolism, to reduce metabolic disorders and a lipid accumulation caused by a sleep restriction.

### 3.3. Metabolite Changes Correlated with Gut Microbiota

Considering that the gut microbiome is a complex bacterial community where interactions between microbes and fecal metabolites have essential functions, we explored their relevance during CISR [33,72]. The functional prediction of the gut microbiota revealed several functions enriched by CISR, for example, the sphingolipid metabolism and fatty acid degradation, which were indicated by the KEGG pathway analysis as well (Tables S3 and S5). The conjoint analysis indicated that the key metabolic pathway-related metabolites were positively correlated with *Parasutterella* (phylum *Proteobacteria*). Several studies have revealed that changes in *Proteobacteria* are closely related to the amino acid metabolism, and the changes in the amino acids can considerably affect the composition and the interactions between the microbial communities [73,74,75]. Consistent with this, we found changes in the histidine and tyrosine metabolism, and a significant positive correlation between anserine and *Parasutterella. Parasutterella*, a genus of Gram-negative bacteria, has been shown to be negatively correlated with high-fat diet obesity and metabolic dysfunction [76]. A previous study suggested that mice with a higher abundance of *Parasutterella* had higher yields of SCFAs and lower incidences of adipogenesis [77]. In this study, we observed elevated levels of lipid metabolites and a lower abundance of the genus *Parasutterella* in CISR mice, indicating that the gut metabolism of CISR mice was affected and the incidence of adipogenesis was higher.

**Figure 8:**
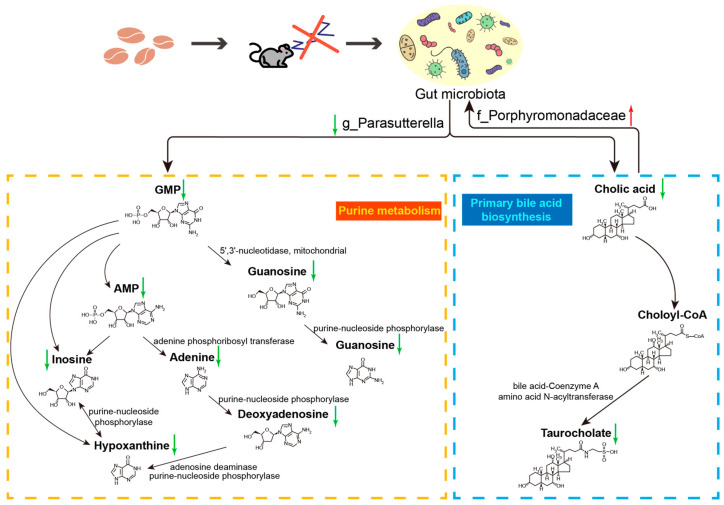
Differential metabolic pathways associated with g_*Parasutterella* and f_*Porphyromonadaceae* on host metabolism after CISR. The composition of g_*Parasutterella*, which was a key factor affecting purine metabolism and primary bile acid biosynthesis, was significantly reduced after CISR. Concomitantly, the abundance of f_*Porphyromonadaceae* was significantly increased, which was affected by bile acid. All enzyme and metabolite names were obtained from the KEGG database.

We also investigated the discrepant metabolic pathways associated with different levels of gut microbiota and found that g_*Parasutterella* and f_*Porphyromonadaceae* were closely related to the purine metabolism and primary bile acid biosynthesis (Figure 8). Previous studies have suggested that *Parasutterella* regulates a variety of differential metabolites in the purine metabolism and primary bile acid biosynthesis, and actively participates in the bile acid metabolism and homeostasis [78]. Purine-derived metabolites have been reported to modulate immune responses and the gut mucosal barrier [79,80]. We found a significant positive correlation between *Parasutterella* and guanine, by which CISR may have potentially adverse effects on the homeostasis of the gut mucosa. Furthermore, bile acid components have been found to exhibit diurnal fluctuations in humans, and disrupting sleep or circadian rhythms has been demonstrated to impact levels of bile acids and their secretion in mice model [81,82]. The results of this study suggest that *Parasutterella* affects the bile acid metabolism and has a positive correlation with cholic acid. A decrease in bile acids appears to favor the overgrowth of *Porphyromonadaceae*, which is one of the pathogenic and pro-inflammatory members of the microbiome, and this may lead to a further down-regulation of bile acid synthesis in a positive-feedback mechanism via inflammation [83]. However, our current data does not demonstrate such a causality, and additional work is needed in future studies to reveal the relevant mechanisms.

## 4. Materials and Methods

### 4.1. Mouse Maintenance and Ethics Statement

C57BL/6N mice (purchased from the Jackson Laboratory, Bar Harbor, ME, USA, 6 weeks of age, male, body weight 20 ± 3 g) were housed in static microisolator cages under environmental conditions of 22 °C, 40–70% humidity, and 12 h:12 h light:dark cycles with the lights on from 9:00 to 21:00. All the mice were fed with standard laboratory rodent feed and filtered water under clean conditions in the same room. All experimental protocols were reviewed and approved by the Animal Care and Ethics Committee of the School of Life Sciences, Lanzhou University (Ethics approval No. EAF2019030). All efforts were made to minimize the animal’s suffering and to use the minimum number of animals required for the acquisition of reliable scientific data.

### 4.2. Sleep Restriction in Mice Model

Fourteen mice were randomly assigned to the CTRL or CISR group, and then were placed in the same type of chamber for 1-week acclimation before the experiment began. To simulate the regular schedule of humans under day-night cycles, mice in both the CTRL and CISR groups were allowed to sleep exclusively during the day (their sleep stage) for 4 weeks. To keep awake of mice during their active period, the automatic sleep deprivation systems (SANS-SA109) were used during the night (21:00–9:00) for both the CTRL and CISR groups. During this 4-week period, the mice in the CISR and CTRL groups were, respectively, given caffeine (20 mg/kg, Sigma-Aldrich, St. Louis, MO, USA) or saline at 9:00 each day. According to previous studies, a caffeine intake could increase the locomotor activity of rodents at approximately 3 mg/kg, with a maximal increase at about 30 mg/kg, but the activity could be reduced at higher doses [84]. It was also stated that 150 mg of caffeine is equivalent to the caffeine content of a Starbucks cappuccino [85]. In this study, based on the human habit of a caffeine intake and previous animal studies on sleep deprivation, we selected 20 mg/kg as the caffeine concentration, which is comparable to 2–3 cups of coffee for an adult person [86,87].

### 4.3. Open Field (Spontaneous Activity) Test

Each animal from the CTRL or CISR group was gently placed in the center of an open field apparatus immediately after a saline or caffeine gavage at 9:00, and its behavior was recorded during the subsequent 3 h. Each open field device consisted of a sound-insulation room (25 cm × 25 cm × 31 cm), and a digital camera was placed above the testing room. The total distance, active time, and the movement trajectory of the mice were automatically recorded and calculated by using the Digbehv 2.0 software (TaiMeng BL-420F in China).

### 4.4. Fecal Sample Processing, 16S rRNA Gene Sequencing and Microbial Diversity Analysis

The fecal samples were collected by using clean metabolic cages, quick-frozen in liquid nitrogen, and then stored at −80 °C for both microbiome and metabolomics analyses. A QIAamp DNA stool Mini Kit (Qiagen, Hilden, Germany) was used to extract the DNA from the pellets, according to the manufacturer’s instructions. The resultant DNA was quantified by ultraviolet spectroscopy and stored at −80 °C for further analysis. The DNA was amplified using the universal primers of 27F (AGRGTTTGATYNTGGCTCAG) and 1492R (TASGGHTACCTTGTTASGACTT) to target the full-length 16S bacterial rRNA. Amplicons were purified using the PCR purification kit (Qiagen, Hilden, Germany) and 1 μg of DNA was used to construct the sequencing library using the SMRT bell Express Template Prep Kit 2.0 (Pacific Biosciences, Menlo Park, CA, USA). The raw reads were deposited into the NCBI Sequence Read Archive (SRA) database (Accession Number: PRJNA880702).

The raw sequenced reads were screened to obtain the CCS (circular consensus sequencing) reads using the SMRT Link. The consensus sequences that did not satisfy the length threshold (1200–1650 bp) were discarded. High-quality reads for bioinformatics analysis were performed, and all of the effective reads from each sample were clustered into operational taxonomic units (OTUs) based on a 97% sequence similarity according to USEARCH (version 10.0) by the UCLUST method [88]. Alpha diversity indexes were calculated to evaluate the species richness and evenness. Beta diversity analyses were used by employing PCoA and hierarchical clustering analysis via the unweighted pair-group method with arithmetic mean (UPGMA) to inspect the similarity of the gut microbial community structure among the samples. The microbial community structure in the phylum and genus of different samples were also described via the Qiime software (version 1.8.0). For the gut microbial functional annotation, the 16s rRNA gene sequencing data were compared with the KEGG (Kyoto Encyclopedia of Genes and Genomes, http://www.kegg.jp/ or http://www.genome.jp/kegg/, version 99.0, accessed on 23 July 2021) pathway database, by means of the PICRUSt (Phylogenetic Investigation of Communities by Reconstruction of Unobserved States) approach, to predict the metabolic function of the microbiota.

### 4.5. Metabolomics Analysis and Data Processing

A total of 25 mg of fecal sample were weighted to an EP tube, and 500 μL of extract solution (acetonitrile:methanol:water = 2:2:1) containing isotopically labelled internal standard mixture was added. After 30 s vortex, the samples were homogenized at 35 Hz for 4 min and sonicated for 5 min in an ice-water bath. The homogenization and sonication cycle were repeated twice. Then, the samples were incubated at −40 °C for 1 h and centrifuged at 13,000 rpm for 15 min at 4 °C. A total of 400 μL of supernatant was transferred to a fresh tube and dried in a vacuum concentrator at 37 °C. Then, the dried samples were reconstituted in 200 μL of 50% acetonitrile by sonication for 10 min in an ice-water bath. The constitution was then centrifuged at 13,000 rpm for 15 min at 4 °C, and 100 μL of supernatant was transferred to a fresh glass vial for LC/MS analysis. The quality control (QC) sample was prepared by mixing an equal aliquot of the supernatants from all the samples. The LC-MS/MS analysis was performed as previously described [89]. The raw data have been deposited into MetaboLights (ID: MTBLS5954). The MS raw data (wiff) files were converted to the mzXML format by ProteoWizard, and processed by the R package XCMS (version 3.2) [90]. The MS2 database was applied for a metabolites preliminary identification [91]. The metabolites were screened by the HMDB (http://www.hmdb.ca/, accessed on 28 January 2021) and KEGG database to obtain the metabolites for analysis.

### 4.6. Statistical Analysis

The difference in the gut microbiome composition was assessed using a non-parametric test and meta analysis via RStudio (version 4.0.2). Normalized data were used for a principal component analysis (PCA), to build orthogonal projections to a latent structures-discriminant analysis (OPLS-DA) model, and consequently to get the variable importance in projection (VIP) value of each metabolite. A 95% confidence interval (CI) was used as the threshold to identify potential outliers in all the samples. For differential metabolite screening, we set a *p* value < 0.05, an FC > 1.2, and a VIP > 1 as the screening condition. The distance measure was set as Euclidean, the clustering algorithm was set as Ward, and the scale was standardized with the abundance of each metabolite. Metaboanalyst 5.0 was used to subject the differential metabolites (*t*-test, *p* < 0.05) annotated by the KEGG pathway analysis, where the background set was set as Mus musculus (mouse), and then the RStudio (version 4.0.2, ggplot2 package version 3.3.2) was used to complete the data visualization. The correlation matrix between the gut microflora-related metabolites and gut bacterial species was generated using Pearson’s correlation coefficient.

## 5. Conclusions

The present study explored the effects of a sleep restriction caused by the consumption of caffeine prior to the sleep period on the intestinal microbial diversity and metabolism of mice, which simulated prolonged wakefulness in humans due to the caffeine intake. Our results suggest that even through caffeine has been demonstrated to exert positive effects on gut health, the sleep disturbances caused by consuming coffee at an inappropriate time may disrupt the gut homeostasis and metabolic equilibrium. In addition, functional changes in the gut microbiota might be the driving factors of the observed changes in the fecal metabolism in response to CISR. Given that our samples only involved male mice, more extensive and intensive studies are still required to investigate whether similar effects can also be observed in females and with other concentrations of caffeine regimes. In summary, our findings in this work may not only contribute to a better understanding of the effects of an untimely coffee break on the intestinal microenvironment, but also have potential implications for advocating a reasonable working and rest schedule.

## Data Availability

The raw reads of 16S rRNA gene sequencing were deposited into the NCBI Sequence Read Archive (SRA) database (Accession Number: PRJNA880702). The raw data of Metabolomics have been deposited into MetaboLights (ID: MTBLS5954).

## Supplementary Materials

The following supporting information can be downloaded at: https://www.mdpi.com/article/10.3390/ijms232314837/s1.
