# Supplementary material for: Caffeine-Induced Sleep Restriction Alters the Gut Microbiome and Fecal Metabolic Profiles in Mice
Source: Int J Mol Sci. 2022 Nov 27;23(23):14837. doi: 10.3390/ijms232314837 (PMC9737546; doi:10.3390/ijms232314837)
Supplement: Supplementary file 1 [file ijms-23-14837-s001.zip › Supplemental Material.pdf]

Supplemental Materials

Caffeine-induced sleep restriction alters the gut microbiome and fecal metabolic profiles in mice

Zan Song[1], Lin Liu[1], Yanyi Xu[1], Ruofan Cao[1], Xianyong Lan[2], Chuanying Pan[2], Shengxiang Zhang[1]*, Haiyu Zhao[1,3]*

[1] School of Life Sciences, Lanzhou University, No. 222 South Tianshui Road, Lanzhou 730000, China.

[2] Key Laboratory of Animal Genetics, Breeding and Reproduction of Shaanxi Province, College of Animal Science and Technology, Northwest A&F University, No. 22 Xinong Road, Yangling 712100, China.

[3] Lead Contact.

Address for Correspondence:

* Dr. Haiyu Zhao zhaohy@lzu.edu.cn (Tel: +86 18153646061)

* Prof. Dr. Shengxiang Zhang sxzhang@lzu.edu.cn

School of Life Sciences, Lanzhou University, No. 222 South Tianshui Road, Lanzhou 730000, China.

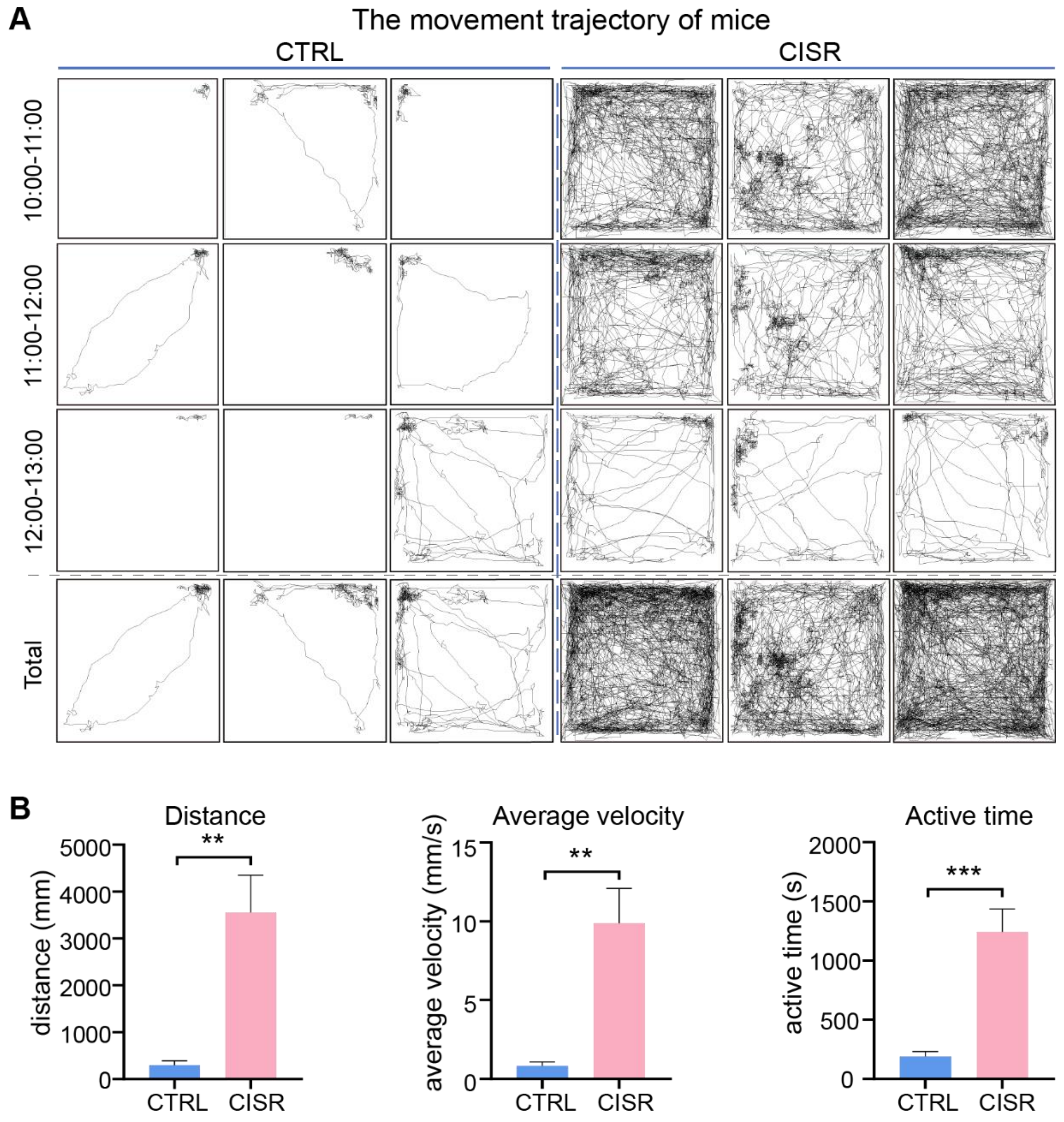

Figure S1. The behavioral verification of caffeine-Induced Sleep Restriction (CISR) model in mice. (A) The representative trajectory traces of CTRL and CISR mice during the three hours after saline and caffeine gavage. (B) The distance moved, average velocity and active time of the CTRL and CISR mice in the open field test after saline and caffeine (20 mg/kg) gavage, respectively. Each experiment was repeated three times independently. Statistical analysis was performed by the T test. n = 12/group. The values are presented as the mean ± SEM. Significant differences are indicated by asterisks (****p* < 0.001, ***p* < 0.01, **p* < 0.05).

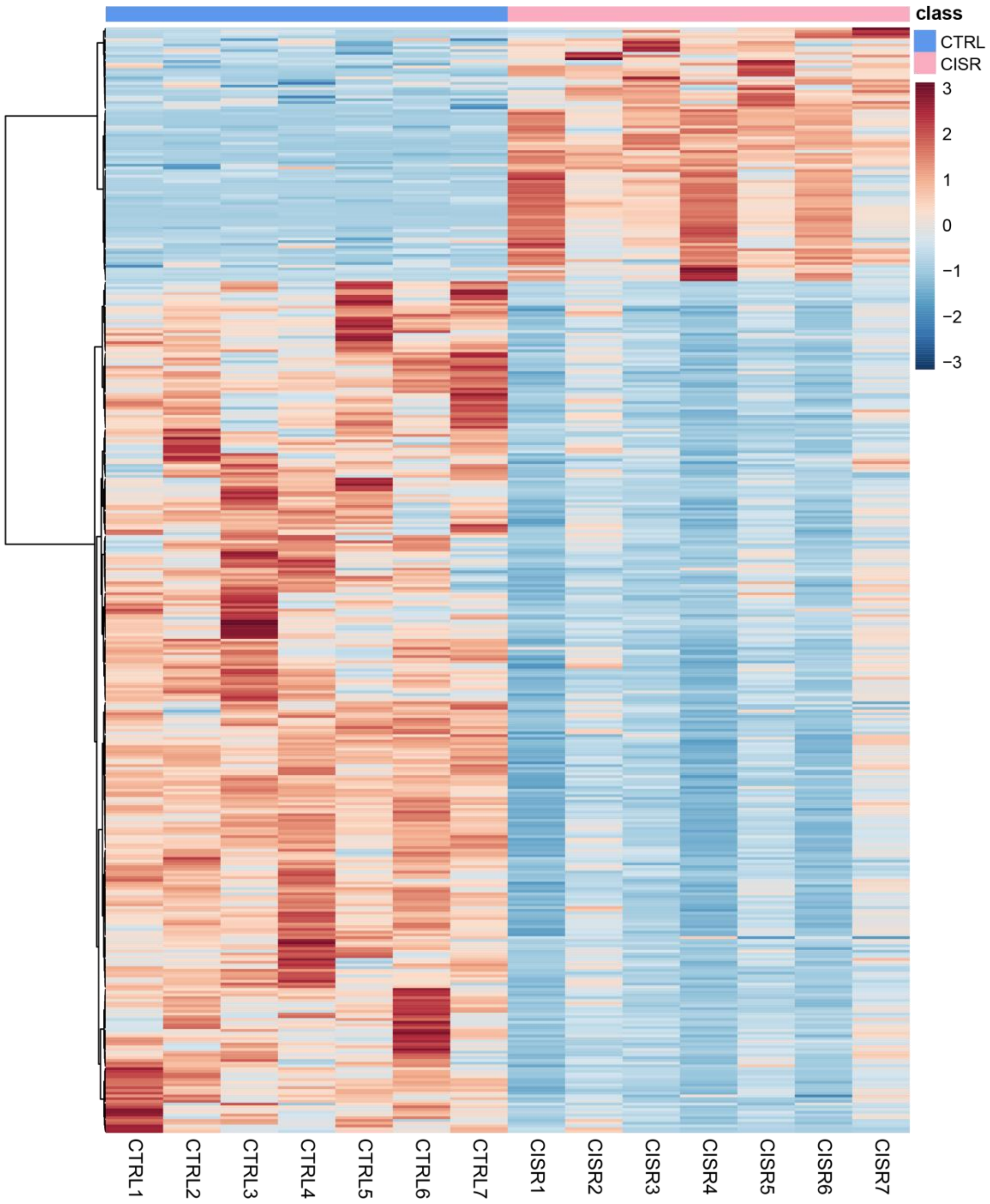

Figure S2. The hierarchical clustering heat map constructed using fecal molecular features with 1.2 fold changes ($p < 0.05$) shows a consistent clustering pattern within individual groups.

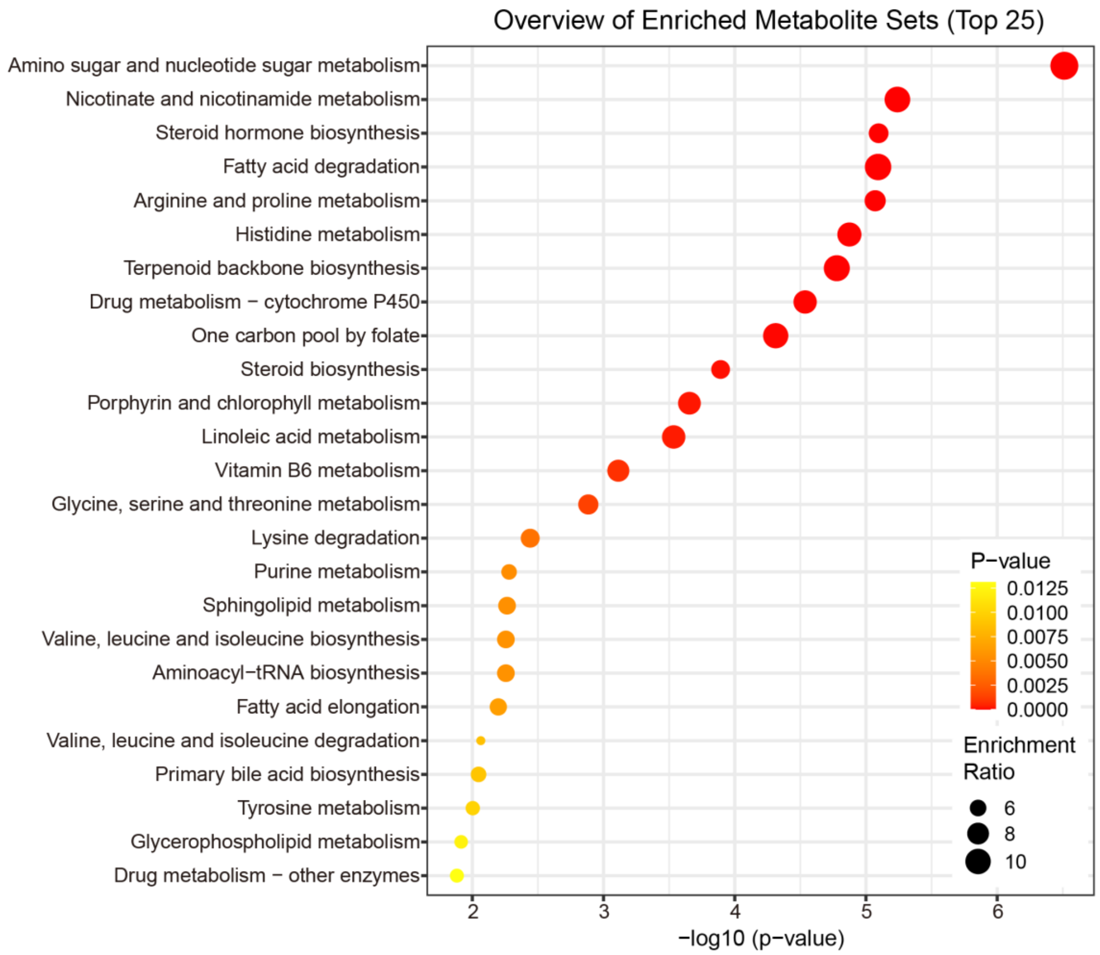

Figure S3. The KEGG enrichment analysis of the dysregulated metabolites ($p < 0.05$, FC > 1.2, compared to controls) to elucidate the correlation between alterations in fecal metabolites and metabolic disorders in CISR mice revealed a close association between the metabolic pathways and the biosynthesis of secondary metabolites.

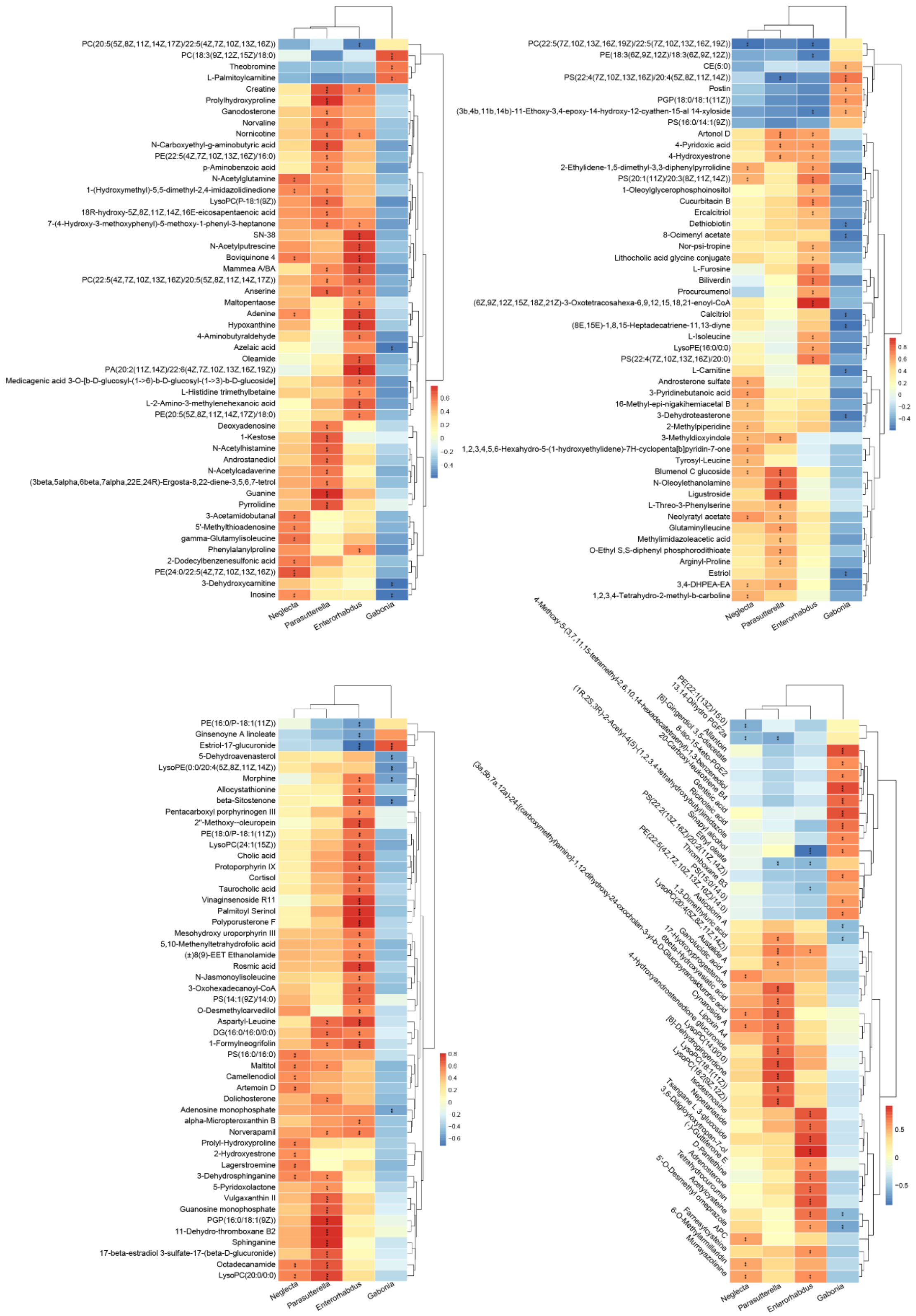

Figure S4. Pearson's correlation analysis between the four genus-level differential microbes and all relevant distinct metabolites ($p < 0.05$) is showed in the heatmap.

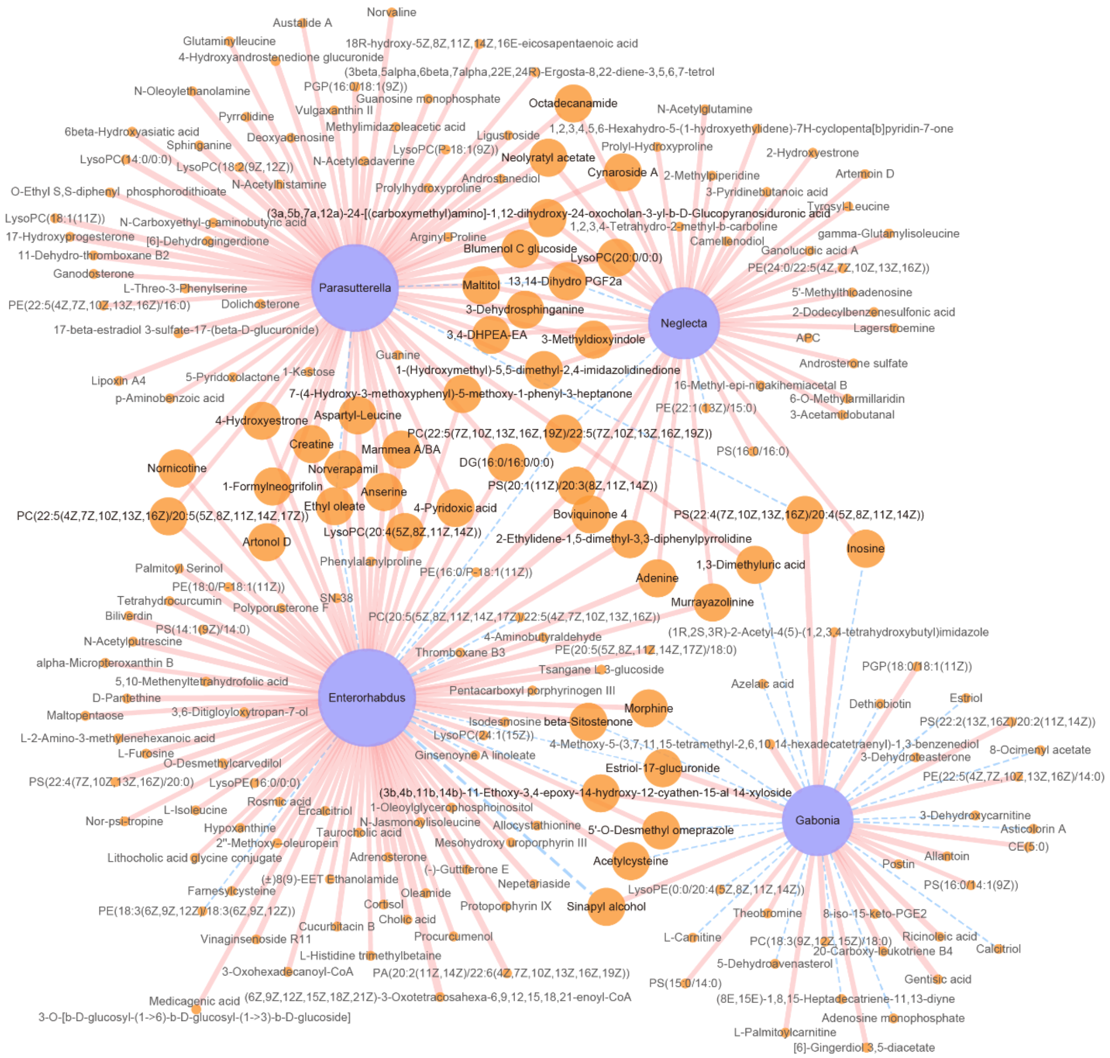

Figure S5. Integrated correlation-based network analysis of the gut microbes and metabolites. Correlation-based network analysis (Pearson's correlation) of genus-level differentially abundant microbes and all differentially abundant metabolites. The size of the nodes indicates the number of significant correlations, and the thickness of lines represents the strength of correlations. Pink solid and blue dashed lines represent positive and negative correlations, respectively.
